# Anti-Dlx5 Retards the Progression of Osteoarthritis through Inhibiting Chondrocyte Hypertrophy and Apoptosis

**DOI:** 10.1155/2022/5019920

**Published:** 2022-03-02

**Authors:** Ye Lu, Chengyuan Zhang, Shilin Jiang, Feng Yuan

**Affiliations:** ^1^Department of Sports Medicine, Shanghai Jiao Tong University Affiliated Sixth People's Hospital, 600 Yishan Road, Shanghai 200233, China; ^2^Department of Orthopedic Surgery, Shanghai Jiao Tong University Affiliated Sixth People's Hospital, 600 Yishan Road, Shanghai 200233, China

## Abstract

Osteoarthritis is a common degenerative joint disease that can cause pain and disability in patients. There is still a lack of effective treatments to improve pathological changes of osteoarthritis cartilages and reverse the progression of osteoarthritis. Our study aimed to investigate the role of Dlx5 in papain-induced osteoarthritis. Osteoarthritis was induced through intraarticular injection of papain. The pathological damage of cartilage tissues was analyzed by H&E staining. The apoptosis of cartilage tissues was detected by TUNEL assay. Immunohistochemical staining was performed to detect DLX5 and BMP-2. Western blot was performed to detect the expressions of SP7, caspase-3, and MYC. The results showed that administration of anti-Dlx5 improved pathological changes of osteoarthritis cartilages, characterized by decreased chondrocyte proliferation, chondrocyte hypertrophy, and matrix damage. Anti-Dlx5 treatment decreased the expressions of BMP-2 and SP7, which are positive regulators of chondrocyte hypertrophy. Moreover, MYC and caspase-3, the critical mediators for chondrocyte apoptosis, were both decreased after anti-Dlx5 treatment. In conclusion, anti-Dlx5 retarded the progression of osteoarthritis by downregulating chondrocyte hypertrophy and chondrocyte apoptosis-related genes. Our findings suggests that Dlx5 is a promising target for osteoarthritis treatment.

## 1. Background

Osteoarthritis (OA) is a degenerative joint disease characterized by articular cartilage degeneration, synovium inflammation, and subchondral bone sclerosis [1]. Injury to knee joint, inappropriate mechanical stress, and chronic inflammation are all considered to increase the risk of osteoarthritis [2, 3]. Osteoarthritis is a common disease among the aged population and a leading cause of disability. Current treatments for OA, such as analgesics and anti-inflammatory drugs, mainly focus on pain management but cannot retard the progression of the disease [4]. New treatment strategies which can retard or even reverse disease progression are needed urgently.

Although osteoarthritis affects the entire joint, the loss of articular cartilage has been considered the primary change of osteoarthritis [5]. Articular cartilage is made up of extracellular matrix and chondrocytes. Under normal condition, chondrocytes in cartilage are usually quiescent and maintain the matrix in a low turnover state [6, 7]. But in osteoarthritis, quiescent chondrocytes become activated and undergo proliferation and hypertrophic differentiation, characterized by enlarged cell size, enhanced expression of collagen type X, and upregulated expression of proteolytic enzymes, such as matrix metalloproteinase 13 (MMP13) [8, 9]. Proteolytic enzymes expressed by hypertrophic differentiated chondrocytes could degrade collagen matrix and hypertrophic chondrocytes eventually undergoing apoptosis and are replaced by calcification, leading to the degeneration of articular cartilage and is responsible for the progression of osteoarthritis [10].

Distal-less homeobox 5 (Dlx5), a nuclear transcription factor, plays an important role in chondrocyte hypertrophy [11]. Numerous studies have reported that Dlx5 is a positive regulator of chondrocyte hypertrophy. Mutant or knockout Dlx5 results in severe defect in chondrocyte hypertrophy [12, 13]. By contrast, overexpression of Dlx5 accelerates chondrocyte hypertrophy [14]. Moreover, it has been reported that Dlx5 is upregulated in osteoarthritis cartilage [15]. However, whether Dlx5 plays a role in osteoarthritis progression has not been investigated.

In this study, we found that anti-Dlx5 treatment improved papain-induced osteoarthritis. Chondrocyte hypertrophy, chondrocyte apoptosis, and extracellular matrix damage, which were increased in cartilages of osteoarthritis, were all reduced after anti-Dlx5 treatment. Mechanically, anti-Dlx5 treatment decreased the expression of two positive regulators of chondrocyte hypertrophy, bone morphogenetic protein 2 (BMP-2), and Sp7 transcription factor 7 (SP7). Furthermore, MYC and caspase-3, which are critical mediators of cell apoptosis, were both reduced after administration of anti-Dlx5.

## 2. Materials and Methods

### 2.1. Animals

Female experimental New Zealand rabbits (age, 8-9 weeks; weight, 2 kg) were purchased from Shanghai Slac Laboratory Animal. Rabbits were housed in metal cages at room temperature (25 ± 2°C) and humidity (55 ± 15%) with light-dark cycle. All animal experiments were performed in compliance with the guide for the care and use of laboratory animals and were approved by Animal Care Committee (approval no. 2018005).

### 2.2. Induction of Knee Osteoarthritis

Following adaptive feeding for 2 weeks, all rabbits were randomly divided into three groups (*n* = 6 per group): normal control group (NC group), OA group, and OA + anti-Dlx5 group. Rabbits were given 3% pentobarbital sodium through marginal ear vein at the dosage of 1 ml/kg body weight. After anesthetized, rabbits were placed on the operating table in the supine position and the knee joints of the rabbits were flexed slightly and disinfected. The rabbits in the OA and OA + anti-Dlx5 groups were given 0.2 ml of 4% papain and 0.1 ml of L-cysteine through intraarticular injection at day 1, day 4, and day 7 to induce knee osteoarthritis [16]. Meanwhile, the OA + anti-Dlx5 group was i.p. injected with 0.3 ml of anti-Dlx5 once a day. The rabbits in control groups were given 0.3 ml of 0.9% saline. At 9 weeks after the first injection of papain, rabbits were euthanized by injecting a lethal dose of phenobarbital (150 mg/kg) and analyzed.

### 2.3. Histological Analysis

Cartilage tissues of experimental rabbits were dissected and fixed in neutral-buffered formalin. Tissues were then decalcified with ethylene diamine tetraacetic acid (EDTA). After decalcification, tissues were embedded in paraffin, sectioned at 6 *μ*m, and mounted onto glass slides. The sections were then dewaxed with xylene and rehydrated with graded alcohol for hematoxylin and eosin (H&E) staining. Sections were analyzed using the light microscope (Olympus).

### 2.4. TUNEL Assay

The apoptosis of cartilage tissues was detected by TUNEL assay according to manufacturer's instructions. Briefly, cartilage tissues were fixed in neutral-buffered formalin, decalcified with EDTA, paraffin-embedded, sectioned into 6 *μ*m sections, and mounted onto glass slides. The sections were then dewaxed with xylene and rehydrated with graded alcohol. Slides incubated using DNase-free proteinase K and endogenous peroxidase were inactivated with hydrogen peroxide. The sections were incubated with the terminal deoxynucleotidyl transferase/nucleotide mixture at 37°C for 1 h. The sections were analyzed using light microscopy.

### 2.5. Immunohistochemistry

Immunohistochemical staining was performed to detect DLX5 and BMP-2 using the streptavidin-peroxidase complex method. Cartilage tissues were fixed in neutral-buffered formalin, decalcified with EDTA, paraffin-embedded, sectioned into 6 *μ*m sections, and mounted onto glass slides. The sections were then dewaxed with xylene and rehydrated with graded alcohol. Sections were incubated in citrate buffer for antigen retrieval. Hydrogen peroxide (3%) and BSA (5%) were used to block endogenous peroxidase and nonspecific binding of antibodies, respectively. The sections were incubated with anti-DLX5 (Abcam, ab109737) and anti-BMP-2 (Abcam, ab214821) primary antibodies at 4°C overnight and followed by incubation with a secondary antibody for 2 hours at room temperature. The streptavidin-peroxidase complex reagent and DAB solution were applied for visualization of expressions of DLX5 and BMP-2.

### 2.6. Western Blot Assay

Cartilage tissues were lysed with a tissue homogenate machine and radioimmune precipitation assay lysis buffer. Protein samples were separated with 10% sodium dodecyl sulphate polyacrylamide gel electrophoresis and followed by transfer to PVDF membrane. The membrane was blocked with 5% bovine serum albumin. Then, the membrane was incubated with primary antibodies and corresponding secondary antibodies. The protein bands were imaged using the SuperSignal West Pico Chemiluminescent Substrate kit. The antibodies used for Western blot are as follows: anti-SP7 (Abcam, ab209484), anti-MYC (Abcam, ab32072), anti-caspase-3 (Abcam, ab13847), and anti-actin (Abcam, ab8226).

### 2.7. Statistical Analysis

Data were expressed as means ± SEM. One-way analysis of variance (ANOVA) with the Dunnett test was performed to compare the means of different groups by using GraphPad Prism. A *P* value of less than 0.05 was considered significant.

## 3. Results

### 3.1. Anti-Dlx5 Ameliorates Papain-Induced Pathological Damage of Cartilage

**Figure 1:**
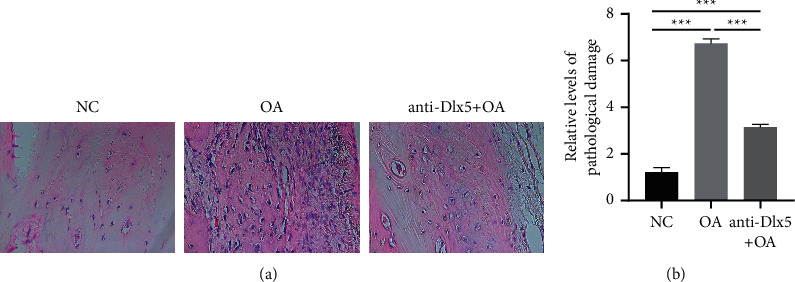
Anti-Dlx5 treatment ameliorates papain-induced pathological damage of cartilage. (a) Representative H&E staining images of cartilages from the NC group, OA group, and OA + anti-Dlx5 group. Scale bar: 50 *μ*m. (b) Pathological damage analysis of H&E staining images of cartilages from the NC group, OA group, and OA + anti-Dlx5 group.

It has been reported that Dlx5 is upregulated in osteoarthritis cartilage [15]. To investigate the role of Dlx5 in osteoarthritis, knee osteoarthritis was induced by intraarticular injection of papain along with cysteine in rabbits. H&E staining showed pathological damage in osteoarthritis cartilage, characterized by increased chondrocyte proliferation, chondrocyte hypertrophy, and matrix damage (Figure 1). The cartilages from the OA + anti-Dlx5 group, however, showed lower extent of chondrocyte proliferation, chondrocyte hypertrophy, and matrix damage than the OA group (Figure 1). These results suggested that anti-Dlx5 treatment ameliorated papain-induced pathological damage of cartilage.

### 3.2. Anti-Dlx5 Treatment Inhibits Chondrocyte Apoptosis

**Figure 2:**
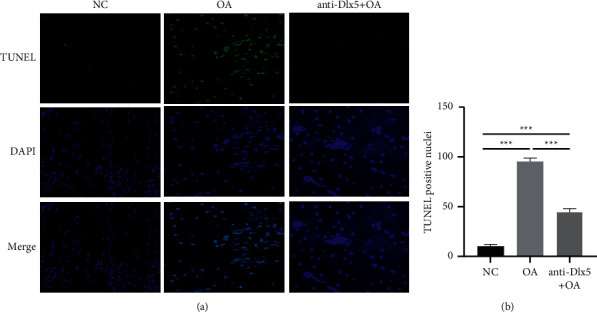
Anti-Dlx5 treatment inhibits apoptosis of chondrocyte. (a) Representative TUNEL staining images of cartilages from the NC group, OA group, and OA + anti-Dlx5 group. Scale bar: 50 *μ*m. (b) The statistical analysis of the number of TUNEL positive nuclei of TUNEL staining images of cartilages from the NC group, OA group, and OA + anti-Dlx5 group.

Chondrocyte apoptosis is a contributor of osteoarthritis progression [17, 18]. In this study, TUNEL assay was performed to analyze apoptosis of chondrocytes. There were increased apoptotic chondrocytes in the cartilage of the OA group (Figure 2), while anti-Dlx5 treatment inhibited apoptosis of chondrocytes as shown by less TUNEL positive cells in the cartilage of the OA + anti-Dlx5 group (Figure 2). These data indicated that anti-Dlx5 treatment may retard papain-induced knee osteoarthritis by inhibiting apoptosis of chondrocytes.

### 3.3. Anti-Dlx5 Treatment Decreases the Expressions of BMP-2 and SP7 in OA Cartilage

**Figure 3:**
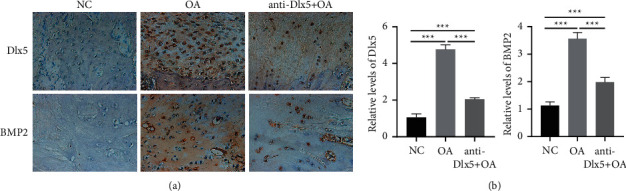
Anti-Dlx5 treatment decreases the production of BMP-2 and Dlx5 in OA cartilage. (a) Representative immunohistochemical staining images of Dlx5 and BMP-2 in cartilages from the NC group, OA group, and OA + anti-Dlx5 group. Scale bar: 50 *μ*m. (b) Quantification analysis of Dlx5 and BMP-2 staining in cartilages from the NC group, OA group, and OA + anti-Dlx5 group.

**Figure 4:**
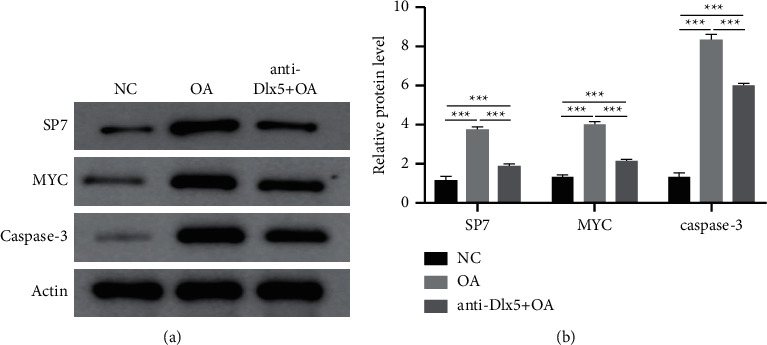
Anti-Dlx5 treatment reduces the production of SP7, MYC, and caspase-3 in OA cartilage. (a) The protein levels of SP7, MYC, and caspase-3 in cartilages from the NC group, OA group, and OA + anti-Dlx5 group investigated by Western blotting. (b) Densitometry analysis of MYC and caspase-3 in cartilages from the NC group, OA group, and OA + anti-Dlx5 group.

Next, we investigated the mechanism by which anti-Dlx5 treatment ameliorated papain-induced osteoarthritis. BMP-2 and SP7 are two positive regulators of chondrocyte hypertrophy and are both reported to be upregulated in osteoarthritis cartilages [19, 20]. Bioinformatics analysis showed that there was a regulatory network among Dlx5, BMP-2, and SP7. The expression level of BMP-2 was analyzed through immunohistochemistry. As shown in Figure 3, BMP-2 was distributed in the extracellular region, which was consistent with its characteristics as a secreted protein. In the cartilage of the OA group, Dlx5 and BMP-2 were both upregulated. Similarly, BMP-2 was downregulated in the cartilage of the anti-Dlx5 +  OA group. These results indicated that BMP-2 may be regulated by Dlx5 and may mediate the osteoarthritis-alleviating effect of anti-Dlx5. The protein level of SP7 was also measured by Western blot. As shown in Figure 4, SP7 was also increased in cartilages of the OA group and decreased after anti-Dlx5 treatment. These results indicated that upregulation of SP7 in osteoarthritis may also be regulated by Dlx5 and involved in the osteoarthritis-alleviating effect of anti-Dlx5.

### 3.4. Anti-Dlx5 Treatment Reduces the Expressions of MYC and Caspase-3 in OA Cartilage

**Figure 5:**
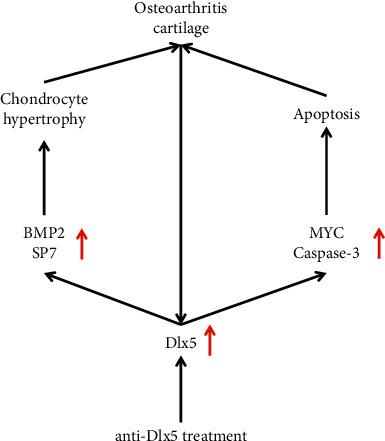
Schematic graph of the mechanism by which anti-Dlx5 retards the progression of osteoarthritis through inhibiting chondrocyte hypertrophy and apoptosis.

Hypertrophic differentiated chondrocytes eventually undergo apoptosis, leading to degeneration of articular cartilage and osteoarthritis progression [10]. Chondrocyte apoptosis was increased in OA cartilages, as shown by TUNEL staining (Figure 5). To validate this process in the molecular level, we detected the protein levels of MYC and caspase-3, which are critical mediators of chondrocyte apoptosis. Consistent with the results of TUNEL staining, the protein levels of MYC and caspase-3 were both increased in OA cartilages and decreased after administration of anti-Dlx5. Collectively, administration of anti-Dlx5 could inhibit the expression of genes related to chondrocyte hypertrophy and apoptosis and thereby retard the progression of osteoarthritis.

## 4. Discussion

Osteoarthritis is a common disease joint disease that can affect any joint, preferentially the knee, hands, spine, and hip. Osteoarthritis can cause lots of pain and disability in patients. Millions of people suffered from osteoarthritis globally [21]. The number of people affected by osteoarthritis is likely to increase due to the aging of the population. Current treatments for osteoarthritis, such as analgesics and anti-inflammatory drugs, mainly focus on pain management but cannot affect the progression of the disease. There is a lack of treatments that can retard or even reverse the progression of osteoarthritis. Here, we found that anti-Dlx5 treatment retarded the progression of osteoarthritis by inhibiting chondrocyte hypertrophy, chondrocyte apoptosis, and matrix damage, suggesting that Dlx5 is a promising target for osteoarthritis treatment.

The benefit effects of medicinal plants have been widely reported in different types of diseases, such as flavonoids, furanocoumarins, coumarins, and emodin in cancers, cannabinoids in gastrointestinal disorders, *Avicennia officinalis* in COVID-19, and *Aglaonema hookerianum* in depression [22]. Dlx5 promotes chondrocyte hypertrophy by upregulating collagen X, vascular endothelial growth factor (VEGF), and matrix metalloproteinases in chondrocytes [23]. Given that chondrocyte hypertrophy can lead to the progression of osteoarthritis, we hypothesized that Dlx5 might play an important role in osteoarthritis progression. Here, we found that Dlx5 treatment indeed retarded osteoarthritis progression by inhibiting chondrocyte hypertrophy and chondrocyte apoptosis. It also regulated two positive regulators of chondrocyte hypertrophy, BMP-2 and SP7.

A previous study has reported that BMP-2 can induce SP7 expression through Dlx5 in myogenic C2C12 cells [24]. By contrast, SP7 overexpression induced the production of Dlx5 in chondroprogenitor cells [25]. Here, we found that Dlx5, SP7, and BMP-2 were all increased in papain-induced knee osteoarthritis, and upregulation of SP7 and BMP-2 were partly dependent on Dlx5. Combined with previous findings, we assumed that there as a regulatory network among Dlx5, SP7, and BMP-2. As a transcription factor, Dlx5 may activate the transcription of SP7 and BMP-2 directly. For instance, Dlx5 can bind and activate the SP7 promoter in myoblasts [26], and the same mechanism may also exist in chondrocytes. The exact mechanism by which Dlx5 regulates SP7 and BMP-2 needs further research. Moreover, TUNEL staining revealed that anti-Dlx5 treatment reduced chondrocyte apoptosis. It has been reported that loss of extracellular matrix induced chondrocyte apoptosis either by initiating apoptotic pathways (e.g., Fas and TNF-*α* receptor) or altering the cytoskeleton, which led to induction of apoptosis [27]. Considering that hypertrophic chondrocytes can degrade extracellular matrix through secreting proteolytic enzymes, we speculate that reduced chondrocyte apoptosis resulted from decreased chondrocyte hypertrophy.

In summary, anti-Dlx5 retards the progression of osteoarthritis by inhibiting chondrocyte hypertrophy and chondrocyte apoptosis. Hence, Dlx5 may be used as a potential target for the treatment of osteoarthritis. The study provides new ideas for the clinical treatment of osteoarthritis. Our findings also indicate that Dlx5 may become a clinical diagnostic marker for osteoarthritis.

## Data Availability

The data used to support the findings of this study are available from the corresponding author upon request.
